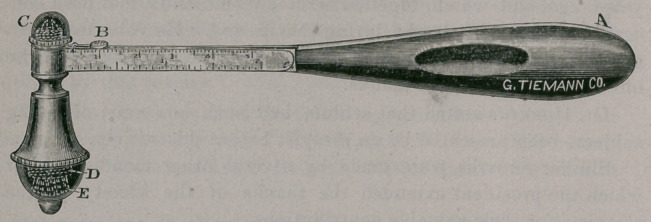# A Neurologist’s Percussion Hammer1Exhibited at the American Neurological Society, Washington, D. C., June 1, 1894.

**Published:** 1895-02

**Authors:** Wm. C. Krauss

**Affiliations:** 382 Virginia Street; Buffalo, N. Y.


### A NEUROLOGIST'S PERCUSSION HAMMER.[1]

BY WM. C. KRAUSS, M. D., Buffalo, N. Y.

THE neurologist requires, as a rule, but very few instruments in conducting an examination of the nervous system, and of such the percussion hammer is most often used. To make the examination thorough and detailed, an office outfit is indispensable, but in the majority of cases, the simplest means are used to ascertain the condition of the sensory and motor conductors, the reflexes, strength of the hands, legs, and other similar conditions.

Having had occasion to make constant use of the hammer in my private and hospital practice, I have devised some improvements and modifications which widen the field of its usefulness, thus making it more serviceable to those engaged in neurological research.

The hammer is constructed after the French pattern, having a heavy metallic head fixed to a flattened oval handle seventeen centimeters long. As a hammer it may be used to examiue the tendon

1. Exhibited at the American Neurological Society, Washington, D. C., June 1, 1894.

and muscular reflexes, to percuss the head, spine, superficial nerves, and the like. The handle (*a*) being of hard rubber becomes warm on friction, while the head being of metal remains cold, thus offering the means of examining the sense of heat and the sense of cold, fulfilling the requirements of a thermo-æsthesiometer.

The cap (*c*) when removed discloses a triangular spear head, about half a centimeter long, while at the other end of the hammer-head is the rounded rubber point; the two ends furnishing, therefore, a sharp and a dull point for examining for anesthesia or hyperesthesia. The advantages of this arrangement over the ordinary way for examining the sensibility of a patient—namely, with a pin or needle, are, that the patient is unaware of the intent of the examiner and does not become hyper-sensitive as soon as the examiner produces a pin; the spear point is not liable to penetrate through the epidermis; the examiner can strike or touch the skin with nearly uniform force, and knowing the sharpness of the point, can better judge whether abnormalities of sensation are present or not.

The spear is divisible into two portions, one securely fixed upon the hammer-head, the other movable upon a metallic slide, upon which is engraved the metric and English scales. This arrangement furnishes an excellent æsthesiometer and is as accurate and convenient as any on the market.

Replacing the cap (*c*) and removing the cap (*e*) a camel's hair brush is exposed, giving a soft surface, while the metallic cap (*c*) gives a hard surface for testing various forms of anesthesia.

It was the intention of the author to have a small ophthalmoscope placed in the handle and then every case of nervous disease, either of the brain, spinal cord or peripheral nerves, could be thoroughly examined with this one instrument.

The hammer is conveniently carried in the pocket and has been of much service to me.

382 Virginia Street.

---

Acute Tonsillitis.—Salicylate of soda is recommended as little less than a specific in acute tonsillitis. It should be given as early in the attack as possible, and in sufficient doses to cause ringing in the ears. Fifteen grains every three hours will usually cause this effect, when the dose may be diminished to ten, and then to five grains at the same intervals. It should be continued a day or two after disappearance of the fever.—*North Carolina Med. Jour.*

## Figures and Tables

**Figure f1:**